# The LET enhancement of energy‐specific collimation in pencil beam scanning proton therapy

**DOI:** 10.1002/acm2.14477

**Published:** 2024-12-07

**Authors:** Blake R. Smith, Daniel E. Hyer

**Affiliations:** ^1^ Department of Radiation Oncology University of Iowa Iowa City Iowa USA

**Keywords:** collimation, LET, PBS, protons, RBE

## Abstract

**Purpose:**

To computationally characterize the LET distribution during dynamic collimation in PBS and quantify its impact on the resultant dose distribution.

**Methods:**

Monte Carlo simulations using Geant4 were used to model the production of low‐energy proton scatter produced in the collimating components of a novel PBS collimator. Custom spectral tallies were created to quantify the energy, track‐ and dose‐averaged LET resulting from individual beamlet and composite fields simulated from a model of the IBA dedicated nozzle system. The composite dose distributions were optimized to achieve a uniform physical dose coverage of a cubical and pyramidal target, and the resulting dose‐average LET distributions were calculated for uncollimated and collimated PBS deliveries and used to generate RBE‐weighted dose distributions.

**Results:**

For collimated beamlets, the scattered proton energy fluence is strongly dependent on collimator position relative to the central axis of the beamlet. When delivering a uniform profile, the distribution of dose‐average LET was nearly identical within the target and increased between 1 and 2keV/μm within 10 mm surrounding the target. Dynamic collimation resulted in larger dose‐average LET changes: increasing the dose‐average LET between 1 and 3keV/μm within 10 mm of a pyramidal target while reducing the dose‐average LET outside this margin by as much as 10keV/μm. Biological dose distributions are improved with energy‐specific collimation in reducing the lateral penumbra.

**Conclusion:**

The presence of energy‐specific collimation in PBS can lead to dose‐average LET changes relative to an uncollimated delivery. In some clinical situations, the placement and application of energy‐specific collimation may require additional planning considerations based on its reduction to the lateral penumbra and increase in high‐dose conformity. Future applications may embody these unique dosimetric characteristics to redirect high‐LET portions of a collimated proton beamlet from healthy tissues while enhancing the dose‐average LET distribution within target.

## 1 INTRODUCTION

Proton therapy treatment planning assumes a constant relative biological effectiveness (RBE) of 1.1 applied throughout the entirety of the calculated dose distribution. The augmented dose distribution is understood to be an approximation; in reality the RBE is closely related to the linear energy transfer (LET) of the radiation, which exhibits an increased ionization density and cell kill probability as the beam penetrates further into the medium. In particular, a rapid increase in LET is observed towards the distal range of a proton beam and across the lateral penumbra due to the exponentially increasing stopping power experienced by low‐energy proton radiation nearing the end of their range.^1^ While the exact relationship between LET and RBE is intensely debated, the importance of studying and modeling the LET with respect to its associated physical dose distribution has been motivated by several recent clinical experiences of adverse treatment side effects attributed to the increased LET of proton therapy implying that a constant RBE approximation is inadequate to model and predict in vivo biological effects.^2^

The advent of pencil beam scanning (PBS) in proton therapy has enabled the delivery of highly conformal treatment plans that are competitive to modern x‐ray volumetric modulated art therapy (VMAT) deliveries.^3^ The lack of exit dose from a particular beam direction makes PBS an appealing option for treatment sites where there are numerous sensitive organs at risk and for the treatment of pediatric patients. However, the proximity of sensitive healthy tissues often near the treatment site increases their risk of injury to high proton LET radiation as high gradients are often intentionally planned to spare nearby tissues from high physical doses. Clinical reports have reviewed cases where changes to the healthy brain parenchyma,^4^ cerebral vasculopathy,^5^ and elevated risk of brainstem necrosis were observed following proton therapy for pediatric patients treated using PBS,^6^ all of which had strong correlations to the variable LET of the proton beam. Similar changes have also been observed among adults treated for a variety of head and neck and base of skull cancers using PBS.^7^ As a result, many clinics and treatment planning vendors offer dosimetric evaluation calculation tools to review LET distributions for a given treatment.

LET has been used to infer RBE within a physical dose distribution for proton therapy from a variety of cell survival data among in silico, in vivo, and in vitro studies.^8^, ^9^, ^10^ These data are applied in clinical practice through models utilizing LET distributions, often calculated using Monte Carlo simulation methods. This is particularly important as underestimating the RBE may result in excessive normal tissue injury and nominally uniform physical dose distributions may be widely heterogeneous due to the corresponding LET distribution. Furthermore, accurate depiction of both calculated LET and its associated biological model is paramount as any planning guided by LET distributions should (1) provide an accurate depiction of LET changes throughout the planning volume and (2) fundamentally represent the appropriate risk of adverse clinical outcomes. Multiple models have been proposed to accurately model the LET using analytical^11^ and Monte Carlo methods^12^, ^13^, ^14^ and have been incorporated as part of the treatment planning process. There are numerous clinical studies that have been published analyzing a treatment plan's LET distribution as a tool to investigate observed normal tissue toxicities following treatment. More recently, prospective use of LET distributions have been proposed to both guide the treatment planning process and directly augment the planning dose distribution using an array of biophysical models to account for the biological impact of high LET regions within the composite physical dose distribution.^12^, ^15^, ^16^

A recent trend in the field of PBS has been towards the development of energy‐specific collimators in PBS as they have been shown to improve lateral conformity and healthy tissue sparing.^17^, ^18^, ^19^, ^20^ The need for these advanced collimation techniques becomes increasingly apparent for shallow targets where the size of an incident beamlet grows considerably as a result of an increasing scatter cross section. Numerous studies have investigated the clinical utility of energy‐specific collimation in PBS and have shown that a substantial improvement to nearby healthy tissue sparing is achievable over uncollimated and aperture‐collimated modalities.^21^, ^22^, ^23^, ^24^ To develop these computational tools, significant effort has been put forth in benchmarking measured physical dose distributions against computational models.^24^, ^25^, ^26^ In particular, Monte Carlo methods have played an essential role in studying these systems as both the fluence and energy spectrum are altered as a result of collimation.^27^, ^28^, ^29^, ^30^

A prominent feature of individually collimated proton beamlets is an increase to the entrance plateau region along the integral depth dose profile. Recent experimental and Monte Carlo studies of the dynamic collimation system (DCS) for both conventionally and spatially fractionated “GRID”‐collimated distributions depict a strong energy dependence that varies with respect to the proximity of the collimating components to the central axis of the beamlet, which has since been described as contaminant scatter from the trimmers.^25^, ^27^, ^28^ Treatment planning of the dose distribution from these collimated dose profiles remain purely physical; no further biological modifications have been considered and the resultant RBE within this region, as well as throughout the entirety of the dose distribution, is regarded as constant.^31^ However, it is likely that this low‐energy component could affect the LET characteristics of the resultant dose distribution in this unique situation. Therefore, it was the purpose of this work to investigate the LET characteristics of the scattered proton radiation as a result of collimation and evaluate the impact that the changes in their spectral signatures may have on a resultant dose distribution through simulating their LET profiles.

## 2 METHODS AND MATERIALS

### 2.1 Monte carlo modeling

Monte Carlo simulations were performed using the Monte Carlo toolkit Geant4 (Version 4.1005)^32^. Unless otherwise stated, the standard g4h‐phy_QGSP_BIC_HP hydronic and g4em‐standard_opt4 electromagnetic transport physics package was used for all charged particle transport in this work. Absorbed dose, fluence, and energy were calculated in water using the standard G4Primitive Scorers. Primitive scorers were written to calculate the track and dose‐averaged LET following the methods of Guan et al.,^33^ where the specific quantity assumed reflects the stochastic lineal energy definition. During the tracking process, the energy deposition, ε, squared energy deposition per step, $\varepsilon^{2}/l$,and path length, l, from each step, i, was tallied for each sensitive volume. The obtained spectrum of these quantities was normalized across all simulation histories, n, to calculate the track, ${\text{LET}}_{t}$, and dose‐averaged, ${\text{LET}}_{d}$, quantities as follows:

(1) $${\text{LET}}_{t}=\frac{\sum_{i=1}^{n}\varepsilon_{i}}{\sum_{i=1}^{n}l_{i}},$$

and

(2) $${\text{LET}}_{d}=\frac{\sum_{i=1}^{n}\varepsilon_{i}^{2}/l_{i}}{\sum_{i=1}^{n}\varepsilon_{i}}.$$

No additional restrictions outside the default transportation parameters were placed on the particle step limit during transport as no small targets measuring less than 500μm were studied; the minimum tally size used in this work was 1mm×1mm×1mm, which is reflective of the minimum voxel size used for treatment planning and are not expected to result in a step size limit dependence.^33^

A custom divergent point source was modeled after the Ion Beam Applications (IBA) Dedicated Nozzle (DN) Proteus Plus beam line at the Miami Cancer Center (MCI) in Miami, Florida. Spot divergence was modeled following the Monte Carlo techniques of Smith et al.^28^, ^34^ and Gelover et al.^25^ by back‐projecting measured fluence distributions along the central axis and calculating an angular fluence pattern at the effective source position. Beam line‐specific parameters that have been experimentally benchmarked against physical dose distributions for the IBA DN system were adopted based on the dynamic collimation monte carlo (DCMC) package from Nelson et al.^27^ and accurately models the double‐focused alignment of two sets of orthogonal nickel trimmer blades to each scanning magnet.^35^ The DCMC package models the center of the *X‐* and *Y‐*trimmers of the DCS at 176 and 213 cm, respectively, from the *X*‐scanning magnet and 171.85 and 208.85 cm downstream of the *Y‐scanning* magnet. Using these models, the beamline optics are mathematically accounted for in the source definition and have achieved excellent spot profile and IDD agreement on and off axis.^27^, ^35^

### 2.2 Investigations of LET distributions

#### 2.2.1 Spectral changes

Spectral changes to the incident beam energy due to collimation were evaluated for 90, 120, and 150 MeV proton beamlets with vary trimmer offset distances defined as the distance from the medial edge of a trimmer blade to the nominal beam spot's central axis. Uncollimated and collimated beamlets were simulated with collimator offsets varied between 0 and 10 mm from the beamlet's central axis for the *X*1 and *Y*1 trimmers separately, combined, and together with their *X*2 and *Y*2 counterparts sharing a common trimmer offset. The later of these scenarios has been studied in the context of improving spatially fractionated (GRID) therapy for PBS^36^ and will be referred to as a *GRID*‐collimated pencil beam. Particle energy and track‐average LET distributions were tallied across a plane 5cm below the *X*‐collimating trimmers using histogram instantiated from the analysisManager class. A total of 1E6 histories were simulated for each scenario resulting in an approximate 1% error at the spectral energy peak. A bin width of 0.1MeV and 0.1keV/μm was used for the kinetic energy and ${\text{LET}}_{t}$, respectively. Spectral features were analyzed from the simulated spectra including the mean, 1st and 99th percentile values across the tails of the spectrum. The cumulative abundance of low‐energy scatter was estimated from the integral portion of the simulated energy spectrum below a threshold from the simulated energy. This relative abundance of the total fluence was quantified as the beamlet's *scatter fraction* where the threshold was set to three standard deviations below the mean of the Gaussian distribution used to model the beamlet's incident energy spectrum.

#### 2.2.2 Per‐beamlet ${\text{LET}}_{d}$

Three‐dimensional absorbed dose‐to‐water and ${\text{LET}}_{d}$ profiles were simulated for individual beamlets distributions using a 1mm×1mm×1mm spatial resolution. Simulations were run for uncollimated and collimated beamlets using $1\times 10^{7}$ particle histories resulting in simulation errors within 1% along the central region of the Bragg peak (BP). ${\text{LET}}_{d}$ and physical dose tallies were post‐processed using a median filter with a one‐voxel span from the ${\text{LET}}_{d}$ simulations and a three‐dimensional Gaussian smoothing function with a 0.5 pixel standard deviation. The lateral profiles were extracted among three energy‐specific depths including the reference depth used for beam output calibration, the BP and half BP depth. Integral depth distributions and lateral cross planes were analyzed at the corresponding simulated BP depths (6.4, 10.65, and 15.75 cm), half BP depths (3.2, 5.32 and 7.87 cm), and clinical calibration depths (2.0, 3.0, and 5.0 cm) for 90, 120, and 150 MeV beamlet energies, respectively. At each depth, in‐plane and cross‐plane ${\text{LET}}_{d}$ profiles were evaluated along each major axis centered along the beamlet's centroid for a particular depth. Along each major axis, the mean ${\text{LET}}_{d}$ and the corresponding ${\text{LET}}_{d}$ along the beamlet's lateral penumbra that coincided with the 10% physical dose level were recorded.

#### 2.2.3 Per‐field LET

Composite‐field ${\text{LET}}_{d}$ dose distributions were calculated for two planning cases consisting of a 4cm×5cm×5cm uniform rectangular prism and an inverted pyramidal target. Collimated treatment plans' collimator positions were forward‐planned using a static set of collimator positions at each energy layer for the cube target and an expanding collimating aperture opening set to the target width for each energy layer for the pyramidal shape. Each individual beamlet was simulated in Monte Carlo to obtain both a physical dose distribution and ${\text{LET}}_{d}$ distribution within a voxelized matrix where each voxel had a spatial resolution of 1mm along each coordinate direction. Targets were optimized to achieve a uniform dose of 25Gy using 4MeV energy spacing and 0.25cm spot spacing resulting in 1331 and 505 beamlets for the cube and pyramid, respectively. Uncollimated and collimated plans were optimized to maintain an equivalent target coverage while minimizing the dose to adjacent normal tissue. The composite dose distributions were then calculated from the optimized weighted superposition of individually simulated dose and ${\text{LET}}_{d}$ distributions which were recalculated from a weighted composite energy transfer and track‐length distribution map based on the quantities of $\varepsilon^{2}/l$ and ε tallied in Equation (2). RBE was calculated from the ${\text{LET}}_{d}$ distribution using a phenomenological RBE model for proton therapy from McNamara^9^ and Paganetti,^1^

(3) $$\text{RBE}=\frac{1}{2D}\left(\sqrt{{\left(\frac{\alpha }{\beta }\right)}_{x}^{2}+4D{\left(\frac{\alpha }{\beta }\right)}_{0}\left(p_{0}+\frac{p_{1}{\text{LET}}_{d}}{{\left(\frac{\alpha }{\beta }\right)}_{x}}\right)+4D^{2}{\left(p_{2}+p_{3}{\text{LET}}_{d}\sqrt{{\left(\frac{\alpha }{\beta }\right)}_{x}}\right)}^{2}}-{\left(\frac{\alpha }{\beta }\right)}_{x}\right)$$

The fitted polynomials were assumed from McNamara^9^ and where $p_{0}=0.99064,p_{1}=0.35605,p_{2}=1.1012,\text{and}\;p_{3}=-0.0038703$. An α/β value of 3.49Gy was assumed for this testing to reflect similar works utilizing this model for proton RBE^1^, ^9^

## 3 RESULTS

### 3.1 Spectral distributions

**FIGURE 1:**
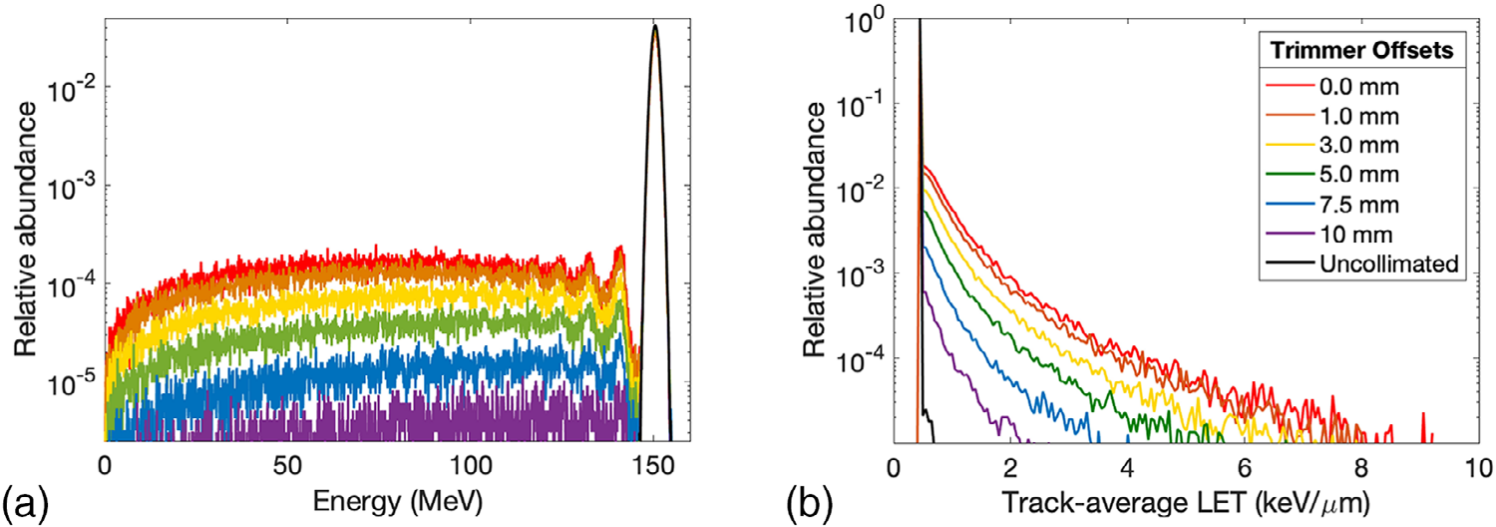
Kinetic energy (a) and track‐average LET (b) spectrum for a 150MeV proton beamlet undergoing differing amounts of collimation by the *X* trimmer. LET, linear energy transfer.

Energy and ${\text{LET}}_{t}$ spectrums that were tallied across the exiting plane of the DCS are shown in Figure 1 for a 150MeV proton beamlet. Changes to the spectral signature can be observed as the collimator approaches the beamlet's central axis as a higher fraction of the total exiting fluence is attributed to low‐energy scatter within the trimmer blade actively collimating the beamlet. The proportion of secondary scatter is nearly uniformly distributed for all energies below the nominal energy distribution of the incident beamlet, changing primarily in its intensity as a function of the amount of collimation that is applied to a specific proton beamlet. This is also reflected in the corresponding ${\text{LET}}_{t}$ spectrum where the maximum ${\text{LET}}_{t}$ increases for increasing amounts of collimation.

**FIGURE 2:**
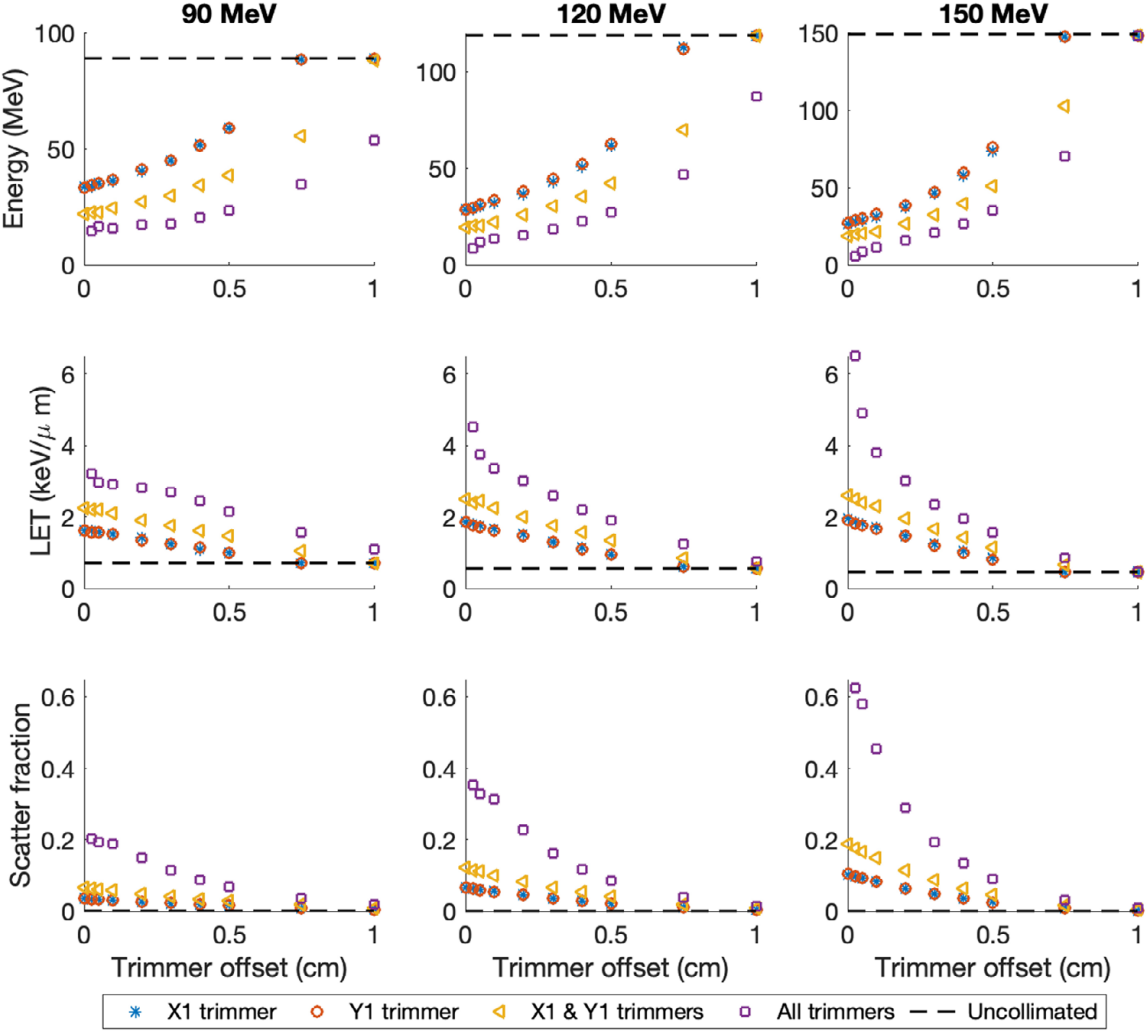
Spectral features calculated from the kinetic energy and track‐average LET (${\text{LET}}_{t}$) spectra for 90, 120, and 150 MeV proton beamlets (columns). (Row 1) The 1st percentile energies from the tail of the kinetic energy spectrum. (Row 2) The 99th percentile LET from the tail of the ${\text{LET}}_{t}$ spectrum. (Row 3) Integral fraction of the total fluence from low‐energy scattered proton radiation from the trimmer. A reference value is plotted using a dashed line to denote the value for an uncollimated beamlet. LET, linear energy transfer.

The majority of spectral changes were observed to occur in the tails of the respective spectra, which are reported for a variety of beam energies and collimation scenarios summarized in Figure 2. Interestingly, no discernible difference was observed in the resulting spectra from either an *X* or *Y* collimator. However, the inclusion of an orthogonal pair of *X*‐ and *Y*‐trimmers positioned to intersect the beamlet along its central axis (0mm trimmer offset) or actively collimating with all four trimmers with a 0.25mm trimmer offset for a 150MeV beamlet increased the scatter fraction by a factor of 1.8 and 6.5, respectively.

### 3.2 ${\text{LET}}_{d}$ along collimated beamlets

**FIGURE 3:**
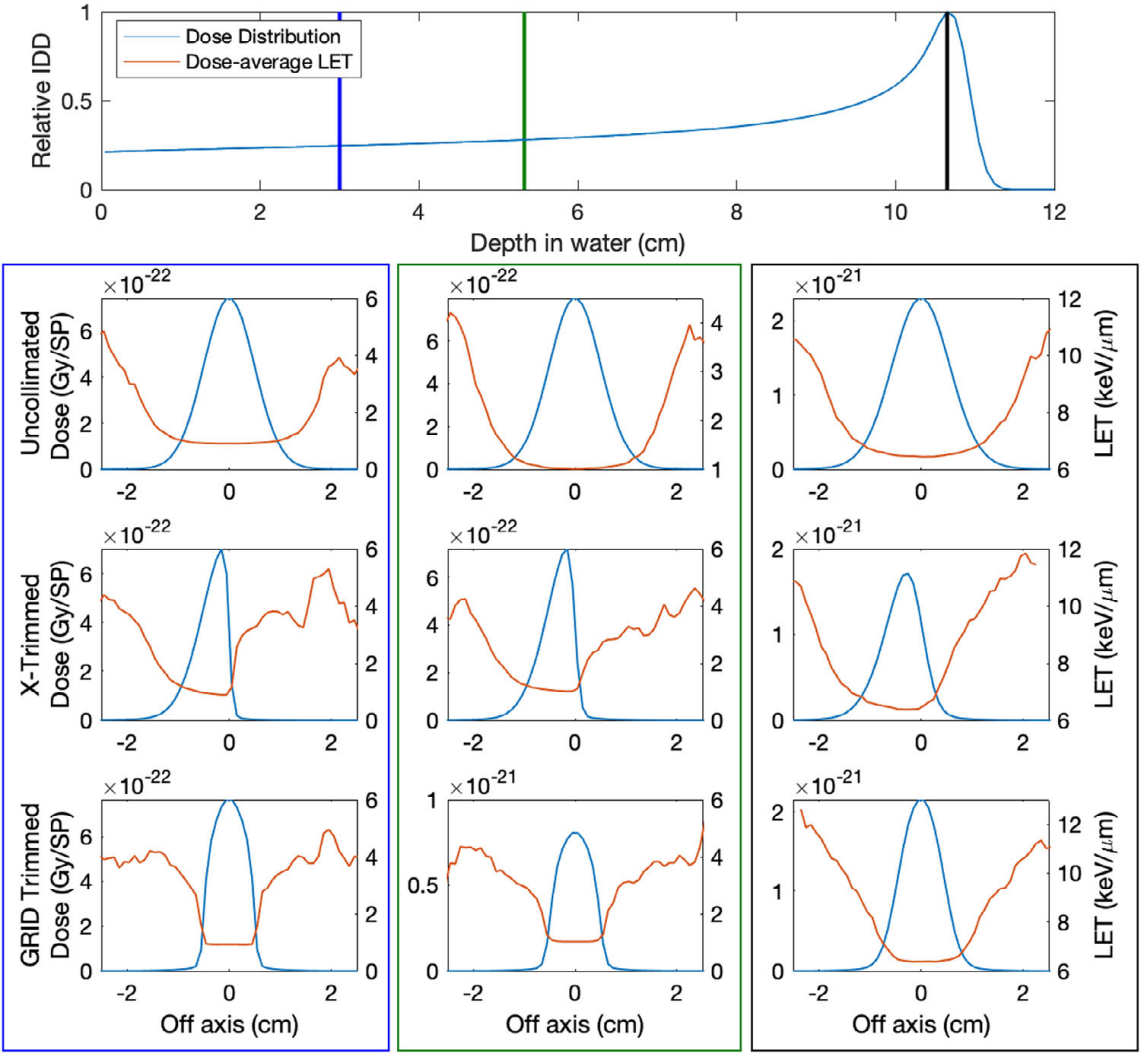
Dose profiles and dose‐average LET profiles at the reference calibration (blue), half (green) and full (black) BP depths for an uncollimated, *X*‐trimmed and GRID‐collimated beamlet collimated using to deliver a 5mm square field. BP, Bragg peak; LET, linear energy transfer.

Figure 3 illustrates the lateral dose and ${\text{LET}}_{d}$ profiles resulting from an uncollimated, single‐trimmed collimated, and for a GRID‐collimated beamlet where all four DCS trimmers share a common trimmer offset for a 150MeV beamlet. For the uncollimated beamlet, the ${\text{LET}}_{d}$ is nearly constant across the entire lateral profile of the spot at the surface. The lateral tails, roughly at 10% of the central peak, start to exhibit an increased ${\text{LET}}_{d}$ relative to the central axis due to the prevalence of large‐angle scatter with increasing depth. In contrast, there is a sharp increase in ${\text{LET}}_{d}$ towards a collimated edge of a proton beamlet while the ratio in ${\text{LET}}_{d}$ between the central axis and the peripheral penumbra is reduced.

Table 1 lists representative ${\text{LET}}_{d}$ values from each analysis depth analyzed for 90, 120, and 150 MeV beamlets. At each depth, a dose‐weighted ${\text{LET}}_{d}$ was calculated by normalizing each voxel's ${\text{LET}}_{d}$ by the dose intensity of the voxel, which was summed across the plane for the entire lateral profile at a specific depth in water. The value given in the parenthesis in Table 1 is the ${\text{LET}}_{d}$ value in the lateral penumbra specified at 10% of the peak dose. While there are penumbra shape differences due to the positioning differences between the *X*‐ and *Y*‐trimmer blades in the DCS, negligible differences were observed between their corresponding ${\text{LET}}_{d}$ profiles. The effect of collimating using all four trimmers resulted in the largest observed increase to the dose‐average LET within the central region of the beamlet. The central axis ${\text{LET}}_{d}$ was nearly identical among all trimmed and untrimmed beamlets at the depth of the BP. Increasing beamlet energy showed an increase to the central axis ${\text{LET}}_{d}$ enhancement between uncollimated and collimated beamlets.

**TABLE 1:** Dose‐average LET (${\text{LET}}_{d}$) (in keV/μm) analyzed at the reference calibration depth, half BP depth, and at the BP for 90, 120, and 150 MeV proton beamlets experiencing differing amounts of collimation from a DCS.

		90MeV			120MeV			150MeV		
	Beamlet	Ref. depth	$\frac{1}{2}$ BP depth	BP depth	Ref. depth	$\frac{1}{2}$ BP depth	BP depth	Ref. depth	$\frac{1}{2}$ BP depth	BP depth
GRID trimmed	Uncollimated	1.14 (1.2)	1.28 (1.36)	7.41 (7.62)	1.01 (1.08)	1.14 (1.18)	6.7 (6.95)	0.99 (1.01)	1.07 (1.05)	6.27 (6.48)
	*X* trimmed	1.24 (2.24)	1.34 (2.11)	7.41 (8.32)	1.17 (1.99)	1.24 (1.58)	6.7 (7.29)	1.16 (1.79)	1.19 (1.33)	6.27 (6.62)
	*Y* trimmed	1.23 (2.18)	1.34 (2.18)	7.41 (8.34)	1.16 (2)	1.23 (1.54)	6.7 (7.23)	1.16 (1.76)	1.18 (1.32)	6.28 (6.69)
	0.25cm	1.58 (2.59)	1.58 (2.17)	7.41 (7.81)	1.5 (2.17)	1.46 (1.67)	6.7 (7.03)	1.41 (1.49)	1.38 (1.44)	6.27 (6.6)
	0.50cm	1.38 (2.5)	1.44 (2.37)	7.41 (8.14)	1.24 (2.08)	1.29 (1.91)	6.7 (7.12)	1.15 (1.63)	1.2 (1.43)	6.27 (6.6)
	1.00cm	1.21 (2.6)	1.33 (1.85)	7.41 (8.02)	1.05 (1.55)	1.17 (1.34)	6.7 (7.05)	1.00 (1.04)	1.08 (1.11)	6.27 (6.59)
	2.00cm	1.14 (1.22)	1.28 (1.36)	7.41 (7.62)	1.01 (1.09)	1.14 (1.2)	6.7 (6.95)	0.99 (1.02)	1.07 (1.08)	6.27 (6.53)

*Note*: Each entry represents the weighted ${\text{LET}}_{d}$ across the entire lateral profile and the corresponding 10th‐percentile ${\text{LET}}_{d}$, listed in parentheses, along the collimated primary axis relative to the beamlet's centroid or average 10th‐percentile among all primary axis for an uncollimated beamlet. Each value is specific to a given depth, and a visual representation of the analyzed profiles is presented for a subset of cases in Figure 3.

Abbreviations: BP, Bragg peak; DCS, dynamic collimation system; LET, linear energy transfer.

### 3.3 Composite dynamically collimated ${\text{LET}}_{d}$ distributions

**FIGURE 4:**
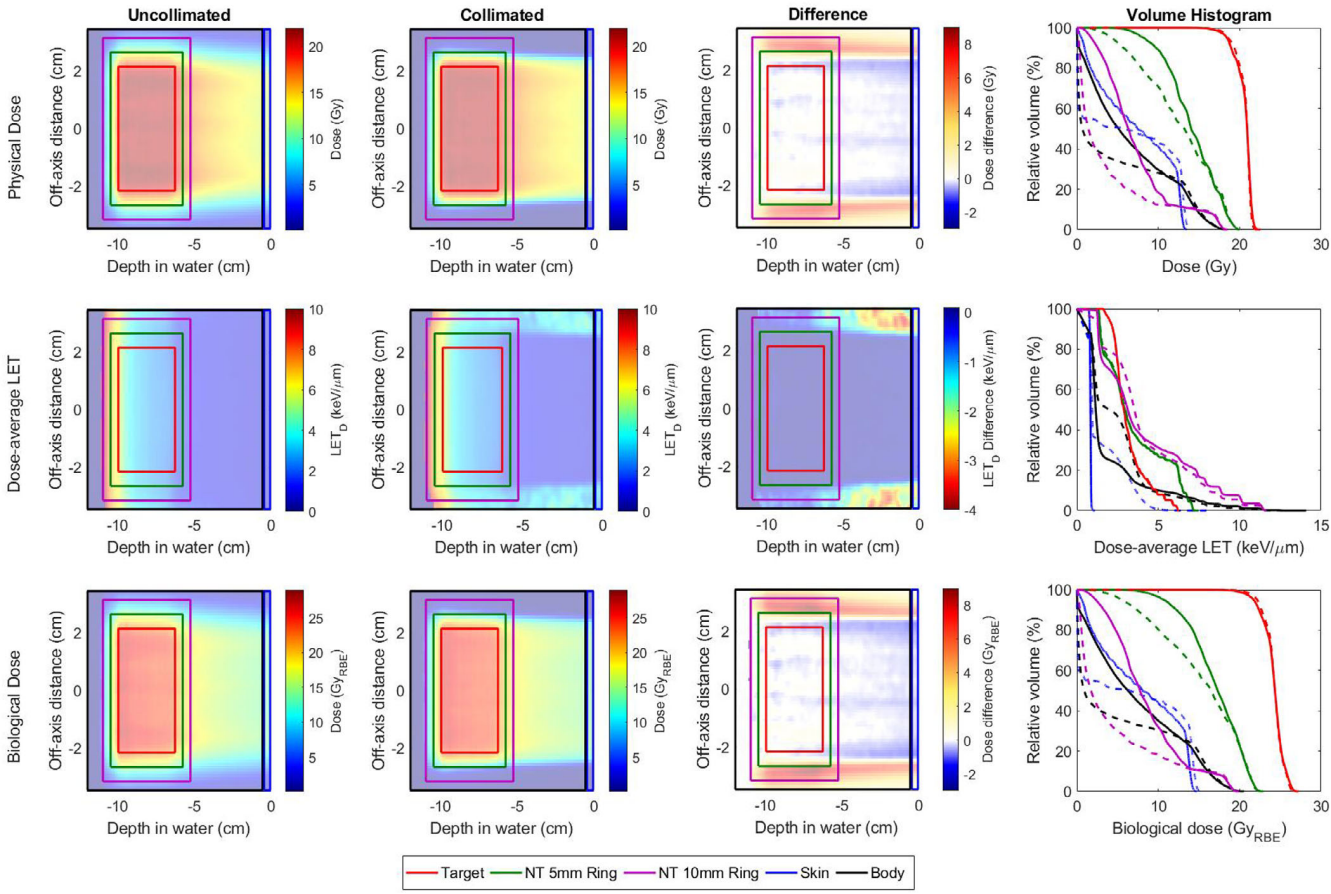
Comparison of collimated (dashed lines) and uncollimated (solid lines) distributions for a rectangular target shape. Collimation was fixed for all energy layers delivered. (Top Row) Physical dose distributions optimized to achieve the desired uniform dose distribution throughout the target. The last column compares the DVH for each delivery. (Middle Row) Dose‐average LET (${\text{LET}}_{d}$) profiles and associated ${\text{LET}}_{d}$‐volume histogram from all regions of interest. (Bottom Row) RBE‐weighted dose profiles for uncollimated and collimated deliveries calculated from their respective LET profiles shown in the middle row. DVH, dose‐volume histogram; LET, linear energy transfer; RBE, relative biological effectiveness.

**FIGURE 5:**
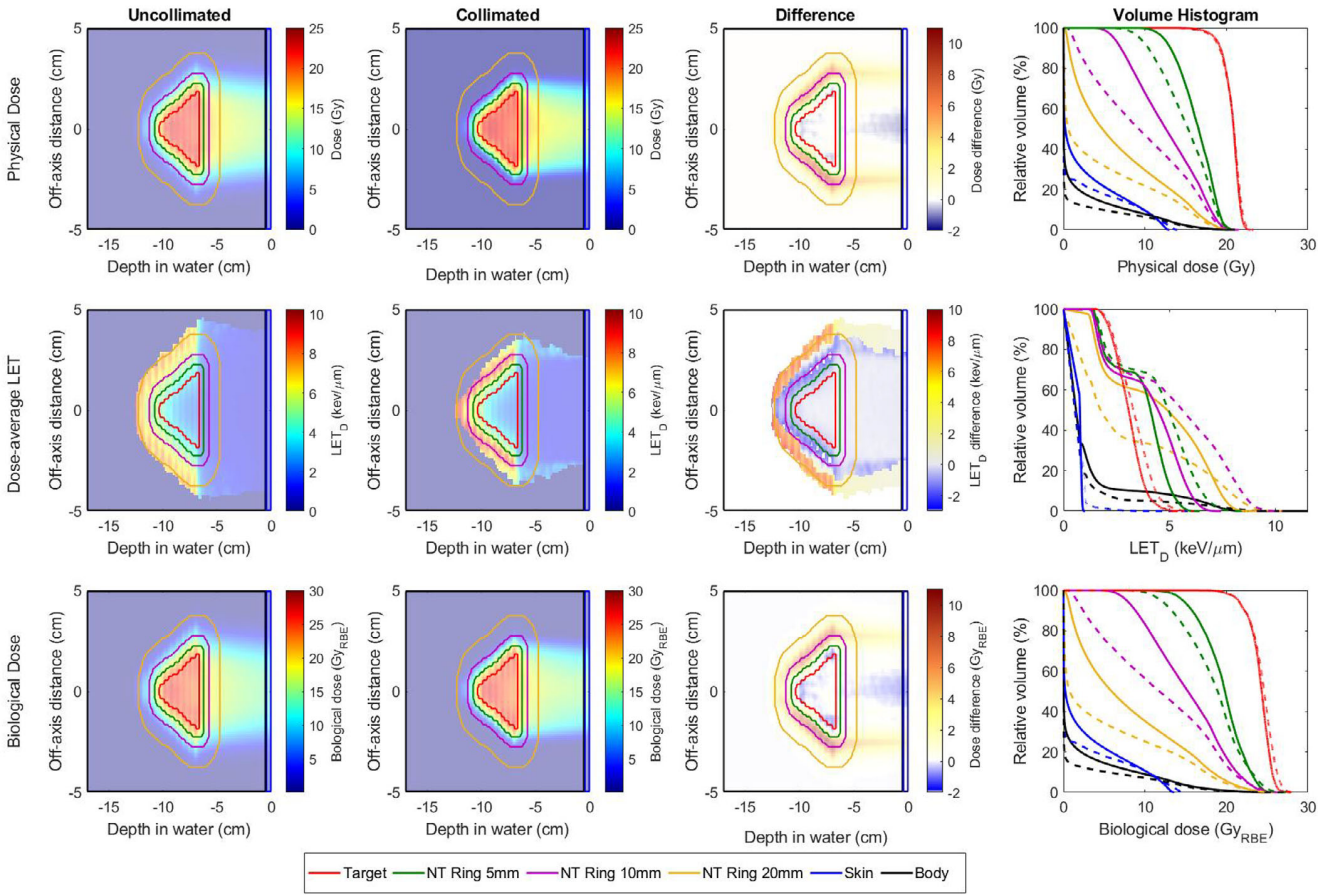
Comparison of collimated (dashed lines) and uncollimated (solid lines) distributions for a pyramidal target shape. Collimation was matched to an expansion of the target boundary for each energy layer. (Top Row) Physical dose distributions optimized to achieve the desired uniform dose distribution throughout the target. The last column compares the DVH for each delivery. (Middle Row) Dose‐average LET (${\text{LET}}_{d}$) profiles and associated LET‐volume histogram from all regions of interest. (Bottom Row) RBE‐weighted dose profiles for uncollimated and collimated deliveries calculated from their respective ${\text{LET}}_{d}$ profiles shown in the middle row. DVH, dose‐volume histogram; LET, linear energy transfer; RBE, relative biological effectiveness.

Treatments were planned for both collimated and uncollimated deliveries using the physical dose distributions. For each case, the resulting ${\text{LET}}_{d}$ and RBE‐weighted dose distribution were calculated and are shown in Figures 4 and 5 for the rectangular and pyramidal shape targets, respectively. Equivalent target coverage was achieved between collimated and uncollimated treatment plans where collimation demonstrated a notable reduction to the surrounding healthy tissue. For the cases presented, the volume containing half prescription were reduced by 18%and25% for the rectangular and pyramidal targets, respectively. The presence of collimation resulted in an increased proximal dose‐average LET beneath and adjacent to the collimator. This is visually apparent for the case of a stationary collimator treating a rectangular target in Figure 4.

For the rectangular target, the ${\text{LET}}_{d}$ distribution was nearly identical within the target for both delivery techniques. However, larger dose differences were observed when evaluating RBE‐weighted dose distributions compared to physical dose distributions between collimated and uncollimated deliveries. In particular, sparing of the healthy tissue from the low‐dose tail appeared to increase for RBE‐weighted distributions when compared against their physical dose distribution as shown in Figure 4. A median dose difference of $4.9\;{\mathrm{Gy}}_{\mathrm{RBE}}\;\;\mathrm{and}\;\;6.0\;{\mathrm{Gy}}_{\mathrm{RBE}}$ was observed for the normal tissue rings 5 and 10 mm from the target.

**FIGURE 6:**
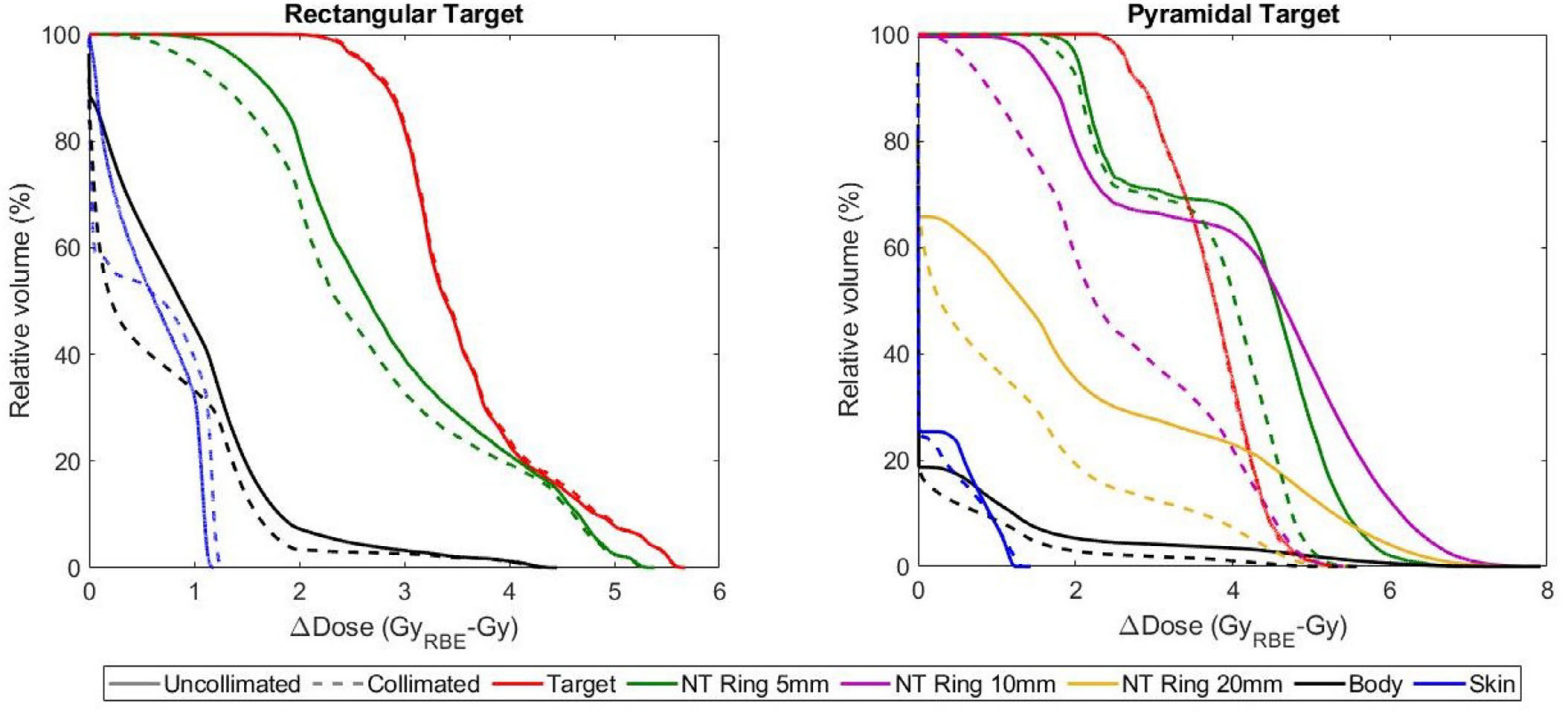
Comparison of collimated (dashed lines) and uncollimated (solid lines) dose difference volume histograms between physical and RBE‐weighted dose distributions for the rectangular and pyramidal targets. RBE, relative biological effectiveness.

The use of energy‐specific collimation for the pyramidal‐shaped target resulted in an increase to the ${\text{LET}}_{d}$ among all structures as shown in Figure 5. In particular, an 11% increase to the upper quartile range of the ${\text{LET}}_{d}$ distribution in the target was observed when collimation was used. Additionally, the increase to the LET experienced within the skin was found to be less than observed for the rectangular targets. Similar to the rectangular target, there was a notable increase to the LET immediately surrounding the target for a collimated treatment while also providing a rapid reduction in the ${\text{LET}}_{d}$ for tissues 10mm or more outside the target, which is reflected in the difference profiles for the physical and RBE‐weighted dose distributions. Table 2 lists the relative changes between DCS‐collimated and uncollimated physical and RBE‐weighted dose profiles for the rectangular and pyramidal targets. The plots shown in Figure 6 quantify the dosimetric differences between the physical and RBE‐weighted dose distributions for collimated and uncollimated deliveries. Similar dosimetric changes were observed when analyzed using RBE‐weighted doses for the rectangular target among all structures for the uncollimated and fixed‐field collimated deliveries, with some small deviations within the low‐dose, high‐volume tail of the body tissue outside the 10mm margin from the target. Specifically, the median change to RBE‐weighted dose to the surrounding normal tissue ring for was 13% larger than for the collimated delivery. These changes were magnified for the pyramidal target which utilized energy‐specific collimation to improve the conformality of the physical dose near the target. While the dose‐average LET was increased in some regions surrounding the 10mm ring, the reduction in lateral penumbra towards the healthy tissue preserved the dosimetric sparing relative to the uncollimated delivery. As shown in Figure 6, the change between the physical and RBE‐weighted median dose to the surrounding 10mm of healthy tissue was 2.05 times larger for the uncollimated delivery.

**TABLE 2:** DVH metrics for the rectangular (rect) and pyramidal (pyr) targets shown in Figures 4 and 5, respectively.

			NT ring median dose				
Target	Delivery	Target $D_{95\;\%}$	5mm	10mm	20mm	Body mean	Skin max
Rect	Unc	1 (1.17)	0.76 (0.94)	0.34 (0.42)	—	0.36 (0.43)	0.73 (0.79)
	Col	1 (1.19)	0.71 (0.89)	0.08 (0.09)	—	0.26 (0.30)	0.75 (0.82)
Pyr	Unc	1 (1.15)	0.89 (1.07)	0.67 (0.82)	0.26 (0.32)	0.10 (0.13)	0.70 (0.73)
	Col	1 (1.17)	0.83 (1.03)	0.50 (0.64)	0.03 (0.04)	0.07 (0.08)	0.76 (0.80)

*Note*: Relative physical doses are listed for each metric with the RBE‐weighted doses included in parenthesis. All dose values are normalized to the physical $D_{95\;\%}$ coverage.

Abbreviations: DVH, dose‐volume histogram; RBE, relative biological effectiveness; NT, normal tissue.

## 4 DISCUSSION

The introduction of collimation in PBS requires special consideration when evaluating its impact on the resultant RBE dose profile. While the ${\text{LET}}_{d}$ is higher in close proximity to the edge of a collimated field, the drastic reduction in lateral penumbra from the field periphery dominates the changes in RBE relative to uncollimated PBS. The incorporation of energy‐specific collimation for the pyramidal target shown in Figure 5 showcases a scenario of how collimating more distal energy layers results in a changing ${\text{LET}}_{d}$ environment across the more proximal energy layers. The ${\text{LET}}_{d}$ volume histogram in Figure 5 shows that the ${\text{LET}}_{d}$ distribution slightly increases throughout the target and to nearby tissues within the 5mm ring while drastically decreasing the ${\text{LET}}_{d}$ in tissues between 1 and 2cm away. These changes resulted in notable differences to the RBE‐weighted dose distribution when comparing collimated and uncollimated treatment plans, which was demonstrated to a lesser extent for a fixed aperture and a rectangular target shown in Figure 4. Such conditions may also hold synergistic potential on a cellular level. A recent investigation into the impact of these ${\text{LET}}_{d}$ changes in the context of radiobiology found that lower DNA damage is expected within the lateral penumbra, which is particularly important for nearby healthy tissues and further suggest that there is the potential for a significant biological benefit even for fixed, per‐field apertures.^37^

Accurate modeling of ${\text{LET}}_{d}$ in collimated PBS will require accurate characterization of the low‐energy scatter emanating from a collimating device. While there exists several publications regarding to experimental validation of physical dynamically collimated dose profiles, no such validation currently exists for dynamically collimated ${\text{LET}}_{d}$ distributions, which will be the focus of future works given the complexity of measuring ${\text{LET}}_{d}$ profiles for proton beams. However, the ${\text{LET}}_{d}$ calculations used in this work are benchmarked against well‐established analytical models and can be used to extrapolate results from our Monte Carlo simulations. The uniform profile distribution shown in Figure 1 suggests that this low‐energy tail is produced from a distribution of scattering experienced by an incident proton near the medial edge of the collimator before exiting the medial side of the collimator. As such, the distribution of these protons would appear to be localized near the upstream medial face of the collimating device resulting in a preferred directivity of the low‐energy scatter in addition to its abundance relative to an uncollimated proton beam. As shown in Figure 3, the low‐energy scatter component appears as a systemic augmentation of the whole beamlet; the impact of the entrance dose is linearly related to the width of the low‐energy tail. As the depth of penetration increases, the influence of the low‐energy scatter on the integral depth dose (IDD) diminishes as conceptually only primaries retain enough kinetic energy to reach the BP. However, the lateral profiles indicate that the changes to ${\text{LET}}_{d}$ distribution persist to this depth and show an enhancement towards the collimated edge. The addition of multiple collimating surfaces increases these observed effects as indicated by the plots in Figure 2 and penumbra features listed in Table 1. A sharp increase in the ${\text{LET}}_{d}$ characteristics occur for small offset distances as well as when additional collimators are used, effectively increasing the number of both low‐energy scatter sources and the abundance of large‐angled scatterers. While there are measurable differences in the physical dose penumbra between the *X*‐ and *Y*‐trimmer blades of the DCS,^27^, ^28^ due to differing geometric locations above the phantom surface, no discernible difference was observed in their respective LET characteristics. While the *X* trimmer is 4.15cm closer to the tally plane, both trimmers are focused to match the divergence of the beamlet and would be expected to have a similar low‐energy scatter spectrum emitted during collimation.

In contrast, some recent studies have observed marginal RBE effects and concluded that the incorporation of collimation has a negligible effect overall due to the high ${\text{LET}}_{d}$ differences from low‐energy scatter from the collimator.^31^, ^37^, ^38^ However, the scope of these recent studies are narrow and focus only on simple, fixed‐field collimators for rectangular apertures covering a rectangular target consisting of a different treatment setup than what would be utilized for the DCS. As shown in the representative uniform field scenario of Figure 4, the majority of the observed differences were located near the superior periphery of the target whereas the dose differences, the same region were there was the greatest difference between uncollimated and collimated treatments when analyzed using physical or RBE‐weighted dose distributions. For this specific case, the increased ${\text{LET}}_{d}$ from scatter off of the collimator is localized as collimation was fixed at the peripheral boundary of the target. Regardless of any contextual differences among ${\text{LET}}_{d}$ models with recent works, it is important to note that collimation continues to provide an irrefutable dosimetric benefit, which is consistent with the conclusions from this work.

## 5 CONCLUSIONS

This work expands upon an existing dosimetric Monte Carlo model to quantify and predict the impact of ${\text{LET}}_{d}$ changes within the treatment region due to the production of low‐energy scatter from collimating devices. The introduction of collimating devices in PBS will result in a spectrum of low‐energy proton radiation that will alter the physical dose and ${\text{LET}}_{d}$ distribution compared to an otherwise unobstructed proton beamlet. Large changes occur to the incident beamlet energy spectrum, primary fluence distribution, and directivity of collimator scatter. These effects are also more pronounced as the beam energy and number of collimating components interacting with the beam increase. In comparison to uncollimated and collimated PBS treatment fields, static apertures for larger irradiation fields are less affected than for smaller volumes undergoing energy‐specific collimation, which exhibit a wider change to the ${\text{LET}}_{d}$ across the treatment volume. The introduction of such low‐energy scatter is a subject of necessary further investigation; it is possible that the ${\text{LET}}_{d}$ distribution can be altered in a manner that would affect RBE calculations. While this often leads to an increase in the RBE‐weighted dose proximal to the target, the introduction of collimation may also serve in some beneficial capacity to direct, and to be optimized from, the expected elevation in ${\text{LET}}_{d}$ near the target periphery.

## AUTHOR CONTRIBUTIONS

**Blake R. Smith**: Conceptualization; data curation; formal analysis; investigation; methodology; validation; writing—original draft. **Daniel E. Hyer**: Conceptualization; methodology; funding acquisition; writing—review and editing.

## CONFLICT OF INTEREST STATEMENT

Dr. Daniel Hyer an inventor on a patent for the DCS that has been licensed to IBA. Dr. Blake Smith has nothing pertinent to this work to disclose.
